# Mixed Emotional Experience Is Associated with and Precedes Improvements in Psychological Well-Being

**DOI:** 10.1371/journal.pone.0035633

**Published:** 2012-04-23

**Authors:** Jonathan M. Adler, Hal E. Hershfield

**Affiliations:** 1 Department of Psychology, Franklin W. Olin College of Engineering, Needham, Massachusetts, United States of America; 2 Department of Marketing, Stern School of Business, New York University, New York, New York, United States of America; King's College London, United Kingdom

## Abstract

**Background:**

The relationships between positive and negative emotional experience and physical and psychological well-being have been well-documented. The present study examines the prospective positive relationship between *concurrent* positive and negative emotional experience and psychological well-being in the context of psychotherapy.

**Methods:**

47 adults undergoing psychotherapy completed measures of psychological well-being and wrote private narratives that were coded by trained raters for emotional content.

**Results:**

The specific concurrent experience of happiness and sadness was associated with improvements in psychological well-being above and beyond the impact of the passage of time, personality traits, or the independent effects of happiness and sadness. Changes in mixed emotional experience preceded improvements in well-being.

**Conclusions:**

Experiencing happiness alongside sadness in psychotherapy may be a harbinger of improvement in psychological well-being.

## Introduction

The respective benefits and drawbacks of positive and negative emotional experience on physical and psychological well-being have been well documented [1]–[3]. Yet, considerably less attention has been given to the ways in which the experience of mixed emotions – that is, the concurrent experience of positive and negative emotions – can affect well-being. A notable exception is the co-activation model of health proposed by Larsen and colleagues [4], which holds that experiencing positive emotions concurrently with negative emotions may detoxify them, transforming a negative emotional experience into fodder for meaning-making and subsequently enhanced well-being. Although recent work has tested the postulates of Larsen's model on physical health [5], very little research to date has directly examined the connection between mixed emotional experience and enhanced psychological well-being. In the present study, we investigated whether mixed emotional experience – specifically the concurrent experience of happiness and sadness – prospectively benefits improvement in psychological well-being. The context for this investigation was a naturalistic longitudinal study of psychotherapy in an outpatient clinic. Psychotherapy is fundamentally concerned with emotional experience [6] and provided an opportunity to assess the unfolding relationships between mixed emotional experience and psychological well-being. The present study aims to demonstrate that concurrent happiness and sadness may temporally precede improvements in psychological well-being in the context of psychotherapy. While this emphasis necessarily restricts the generalizability of this study to the psychotherapeutic context, the current work may have implications for the basic scientific understanding of mixed emotional experience as well as for influencing psychotherapeutic practice.

### The role of mixed emotions in psychological well-being outcomes

When facing negative events in the course of one's life, people may choose to either suppress negative emotions [7] or express them [8]. There are benefits and drawbacks to both approaches, but failing to confront negative events can ultimately lead to increased stress levels [9]. Larsen and colleagues [4] propose that a third strategy, one of “taking the good with the bad,” might actually benefit individuals during difficult times by allowing them to confront adversity and ultimately find meaning in life's stressors (a eudaimonic outcome), as well as to feel better in their wake (a hedonic outcome). In their co-activation model, allowing for the experience of positive emotion alongside negative emotion prompts individuals to face negative life events and gain insight into them.

Larsen and colleagues' [4] model thus suggests that during difficult situations, a mix of positive and negative emotions may be optimal for well-being. For instance, when experiencing the loss of a loved one, allowing positive memories to be experienced alongside sadness could potentially lead to a healthier bereavement process [10]. As Davis and colleagues [11] note, one key to resilience across the adult life span, may be the “ability to maintain affective complexity in the face of life's inevitable difficulties” (p. 1155).

Zautra and colleagues' research on the Dynamic Model of Affect (DMA) [12], [13] also speaks to this relationship. Namely, the DMA holds that when people are feeling calm, they process information from a variety of sources so as to create a balanced and rich assessment of their environments; benefits can be accrued from gaining a full understanding of contexts and experiences. However, when individuals are in the midst of difficult and uncertain situations, the need to process information in a quick and rapid fashion ultimately takes precedence over any advantages that might result from a more nuanced assessment of a situation. In such circumstances, attention narrows so that one can deal with immediate concerns, an idea that resonates with Fredrickson's Broaden-and-Build theory of positive emotions [14]. Generally, the DMA suggests that difficult life situations will tend to cause emotional experience to be much less complex than comparatively less difficult situations. There are, however, individual differences in the extent to which people experience affective complexity in the face of negative situations [12]. Theoretically, just as people experience some degree of affective complexity in calm states to gain a fuller understanding of their environments [11], it stands to reason that those individuals who are better able to maintain this mixture of positive and negative emotion during difficult times will be better able to learn from life's ups and downs, and ultimately show enhanced psychological well-being.

Along these lines, Bonnano and Keltner [15] have found that bereaved adults who expressed positive emotions when talking about their recently deceased spouse (during what was an otherwise negative grieving period) experienced reduced grief over time. Perhaps most directly related to the present research, Coifman, Bonanno, and Rafaeli [16] found that participants who showed a less severe negative correlation between positive and negative emotions also evidenced greater resilience to loss. Furthermore, Ong and colleagues [17] showed that experiencing higher levels of positive emotion on stressful days was related to successful adaptation among widows in later life. Thus, the body of literature on mixed emotional experience outside the clinical context suggests that the concurrent experience of positive and negative emotions is positively associated with psychological well-being. Drawing on previous research that has focused on happiness and sadness specifically [18], [19], including Lazarus' [20] proposal that these are emblematic positive and negative emotions, in the current study we operationalized mixed emotions as a mix of happiness and sadness.

Psychotherapy is fundamentally concerned with emotional experience. Regardless of the specific approach to treatment, all psychotherapies grapple with patients' emotions as they relate to psychological well-being [6]. For example, exposure therapies for anxiety disorders seek to activate patients' negative emotion (anxiety) in controlled experiences in the service of promoting habituation and cognitive restructuring around the meaning of negative emotion [20]. Likewise, positive psychotherapy [21]–[23] suggests that depression can be addressed not only through a reduction of negative emotions, but also through the building of positive emotions, personal meaning, and character strengths. Different approaches to treatment adopt different strategies for eliciting and addressing emotions, but given that the literature on patients' perspectives on treatments suggests that the individuals undergoing treatment are most attuned to those elements of psychotherapy that transcend differences between theoretical orientations [21], [22], we focused on a variety of treatments in a natural context. Thus, the present study sought to examine the associations between mixed emotional experience and psychological well-being in the context of psychotherapy as it is practiced in typical treatment settings.

### What is psychological well-being?

The small group of studies that have specifically investigated the relationship between mixed emotional experience and psychological well-being has implicitly embraced a multi-faceted notion of well-being, including both *hedonic* and *eudaimonic* elements [15], [17]. Ryan and Deci [24] suggest that hedonic conceptions of psychological well-being tend to emphasize three dimensions: life satisfaction, the presence of positive affect, and the absence of negative affect. In contrast, eudaimonic conceptions of psychological well-being emphasize self-actualization and vitality [24], [25]. It is important, though perhaps unsurprising, that mixed emotional experience might be associated with hedonic well-being, given that both are centrally concerned with affect. However mixed emotional experience might also be associated with eudaimonic well-being. In the present study we adopt a holistic operationalization of psychological well-being that taps both hedonic and eudaimonic dimensions. As a result, we employ the more general term, “psychological well-being,” to encompass this approach.

### Overview of present research

Taken together, the studies reviewed above offer preliminary evidence for the positive role that the blending of positive and negative emotion can play in psychological well-being. Yet, none has systematically examined the *prospective* benefits that mixed emotions may have on psychological well-being over time in a fine-grained way. That is, while the above studies showed associations between mixed emotions and improved psychological well-being, none showed that mixed emotional experience (and concurrent happiness and sadness in particular) could be linked to improved psychological well-being in a longitudinal sense or examined the time course of these relationships. Furthermore, the existing research on mixed emotional experience has largely overlooked psychotherapy as a prime natural experiment for investigating the dynamic relationships between mixed emotions and psychological well-being. Thus, in the present study, we sought to examine whether mixed emotional experiences are prospectively linked to enhanced psychological well-being in the context of psychotherapy. Just as these other studies sought to produce results that might generalize beyond their specific focus on the experience of bereavement and loss, in the present study we have focused on psychotherapy as a paradigmatic example of an experience concerned with emotion. In doing so, we attempted to maximize ecological validity by longitudinally tracking a diverse sample of individuals undergoing psychotherapy who were engaged in varied processes of actively coping with adversity [6], [26]–[28]. Although there is a wealth of research on session-by-session change in psychotherapy [29]–[31], this previous work has not specifically examined the complex interplay between positive and negative emotion and its effect on therapy patients' psychological well-being. By examining participants on a session-by-session basis in the present study, we are able to evaluate the role that mixed emotional experience plays in therapeutic change.

We hypothesized that among psychotherapy participants 1) the concurrent experience of happiness and sadness would be associated with higher levels of psychological well-being over time, and 2) the concurrent experience of happiness and sadness would be prospectively linked to increases in psychological well-being.

## Methods

### Participants

Forty-seven adults (*M_age_* = 36 years) who sought treatment at a major outpatient clinic for a wide variety of problems, ranging from significant psychopathology to more typical life events such as divorce or the transition to parenthood, were enrolled in the present study prior to beginning treatment. Participants were eligible to participate if they were over 18 years old and seeking individual (as opposed to couple, family, or group) psychotherapy. There were no other exclusion criteria. Demographic description of the sample of participants can be found in Table 1. Demographic description of the therapists who had patients enrolled in this study can be found in Table 2. The sample was similar to that found in the general treatment-seeking population [32].

**Table 1:** Demographic characteristics of sample (participants).

Category	Sub-Category	Number (% of sample)	M (SD)
**Sex**	Female	33 (70.2)	–
	Male	14 (29.8)	–
**Race**	Caucasian	34 (72.3)	–
	African-American	5 (10.6)	–
	Hispanic/Latino(a)	4 (8.5)	–
	Arab-American	2 (4.3)	–
	Asian-American	1 (2.1)	–
	Multi-Racial	1(2.1)	–
**Age (years)**		–	35.68 (12.99)
**Income**		–	3.25 (1.44)
**Education**		–	5.39 (1.73)

Note:

For Income: 1 = Under $10,000, 2 = $10,000 to $20,000, 3 = $21,000 to $40,000, 4 = $41000 to $60,000, 5 = $61,000 to $100,000, 6 = Over $100,000.

For Education: 1 = Less than high school, 2 = High school/GED, 3 = Some college, 4 = Technical school degree, 5 = Associate degree (2 year college), 6 = Bachelor's degree, 7 = Master's degree, 8 = Professional doctoral degree or equivalent.

**Table 2:** Demographic characteristics of sample (therapists).

Category	Sub-Category	Number (% of sample)	M (SD)
**Sex**	Female	27 (84.4)	–
	Male	5 (15.6)	–
**Race**	Caucasian	24 (75.0)	–
	African-American	3 (9.4)	–
	Asian-American	3 (9.4)	–
	Arab-American	1 (3.1)	–
	Multi-Racial	1 (3.1)	–
	Hispanic/Latino(a)	0 (0.0)	–
**Education**	Current Graduate Student	16 (50.0)	–
	Master's-Level	13 (40.6)	–
	Doctoral-Level	3 (9.4)	–
	Senior Doctoral-Level	1 (3.1)	–
**Theoretical Orientation**	Cognitive-Behavioral	14 (36.8)	–
	Integrative	13 (34.2)	–
	Psychodynamic	8 (25.0)	–
	Emotion-Focused	1 (3.1)	–
	Humanistic	1 (3.1)	–
	Interpersonal	1 (3.1)	–
**Age (years)**		–	30.73 (7.38)
**Patient fee (US dollars)**		–	36.96 (35.40)
**Duration of Therapy**	Number of Assessment Points	–	10.59 (3.03)
	Number of Days	–	103.00 (39.64)

Note: Classification of theoretical orientation was based on therapist self-report to an open-ended probe asking them to describe the theoretical orientation they used with this patient. Many therapists responded to the open-ended probe with one of these labels. In the four instances where therapists instead described the specific techniques they used, an attempt was made to classify the treatment into one of these categories (three were labeled Integrative; one was labeled Cognitive-Behavioral). The distribution of theoretical orientation sums to 38, although there were 32 therapists, because some therapists used different primary theoretical orientations with different patients enrolled in the study.

### Materials

#### Assessing psychological well-being

In order to tap a broad conception of psychological well-being encompassing both hedonic and eudaimonic elements [24], the Systemic Therapy Inventory of Change was selected as the primary outcome measure (STIC) [33]–[36]. The STIC, a self-report questionnaire, is the first and only measure specifically designed to bring a multi-systemic perspective to the study of patient change in individual therapy [35]. The STIC offers several benefits, compared to other commonly used measures, including its holistic assessment of participants' psychological well-being, as well as its systemic perspective which assesses the participant and his or her greater relational context. In the present study, the STIC subscale specifically designed to assess changes in the patient's overall individual psychological well-being (the Individual Problems and Strengths (IPS) subscale) served as the primary index. In both the initial and intersession versions of the STIC this 24-item subscale shows good convergent validity with widely used measures of depression and anxiety [35], [36]. On this subscale, participants respond to questions that tap hedonic well-being, such as “How well have you been getting along emotionally these days” and those that tap eudaimonic well-being, such as “I am comfortable with who I am,” along 5-point scales ranging from 1 to 5, where higher scores indicate more positive well-being.

#### Assessing personality traits

In addition to the STIC, participants also completed the Big Five Inventory (BFI), a widely-used measure of broad dispositional personality traits, two of which – neuroticism and extraversion – have long been shown to be associated with emotional experience [37], [38]. An assessment of personality traits was included in the present investigation in order to ensure that any changes observed in participants' use of mixed-emotional language and their psychological well-being were not simply reducible to dispositional affective tendencies.

#### Assessing emotional experience

To assess the emotional content of participants' experiences, we collected private narratives about participants' perspectives on treatment. The present study asked participants to reflect in writing on their thoughts and feelings associated with being in therapy, including the way they saw the treatment fitting into their overall life or sense of self. As such, the narratives discussed both participants' life events as well as their experiences in treatment. They therefore concerned the way participants were working through the difficulties that brought them into treatment as well as the impact of that treatment on their sense of self. For example, here is an excerpt from one participant whose narrative features both happiness and sadness in discussing her treatment:

I am committed to trying to make every day better than the day before. So far, it's been tough going at times, with frequent setbacks involving much sadness and feelings of helplessness at times. But the fact that I'm working on improving in and of itself makes me feel better about my future and makes me happy and hopeful despite my slow progress and often listless feeling.

In this example, the participant highlights both her sadness and her happiness as she describes the difficult work of therapy. In another example, a different participant describes his mixed emotional experience about the events of his recent life:

This has been a difficult couple of weeks. My wife and I celebrated the good news of a healthy pregnancy report at nine weeks (the time when we lost our pregnancy last January). But I also feel the sadness of still looking for a job and for my wife and my pending loss of my wife's grandmother. It feels as if “what more can I take.” But, in reality I also feel reasonably confident and happy. Not that I don't feel down, but I also feel happy with my marriage.

In this excerpt, the participant notes the concurrent positive and negative emotions he has experienced over the past two weeks. He describes feeling both happy and sad at the same time in the context of his marriage.

This approach to collecting patients' perspectives has been touted as a robust vehicle for tapping the private ways in which individuals understand their experiences in both clinical and community samples [26], [27], [39], [40]. It has been used in several prior investigations of patients' internal process over the course of psychotherapy [39], [41]–[44].

### Procedure

#### Assessment schedule

Prior to the first session of treatment, participants completed the STIC, the BFI, and wrote a personal narrative. Then, in between every session of psychotherapy for the next twelve sessions participants again completed the STIC and wrote narratives providing their perspectives on treatment. After the twelfth assessment point, participants again completed the BFI to assess dispositional traits. Five participants concluded treatment prior to the twelfth assessment point, two concluded treatment at the twelfth assessment point, and the rest continued on in treatment past the twelfth assessment point. This variability was appropriate, given that the focus of the present study was on assessing the unfolding relationships between mixed-emotional experience and psychological well-being, not on assessing final treatment outcome. At all times, narratives were kept confidential from therapists, such that participants could feel free to express their genuine perspectives. In total, over 500 narratives were collected.

#### Blinding narratives

Following the completion of participation for the entire sample, participants' narratives underwent an extensive blinding procedure. They were assigned random study identification numbers and transcribed by individuals not involved in subsequent coding of the narratives. This procedure ensured that once narratives were coded by raters there was no way to identify which individual or which session a given narrative was drawn from.

#### Coding narratives

A team of two trained raters (undergraduate research assistants, trained by the first author, who were blind to the hypotheses of the study and unfamiliar with the coactivation model) coded the narratives for their emotional content. Previous theoretical and empirical work on mixed emotional experience has taken a broad approach to operationalizing the construct, including generic categories of “positive” and “negative” emotional experience. In contrast, in the present study we sought to empirically identify the specific blend of positive and negative emotions that are associated with improvements in psychological well-being. The narratives were coded for eight specific emotions (happiness, excitement, surprise, sadness, fear/anxiety, anger, shame, guilt). These emotions were selected based on the seminal work of Ekman [45] identifying universally expressed emotions (happiness, surprise, sadness, fear/anxiety, anger), and augmented with several additional emotions likely to be relevant in the therapeutic context (shame, guilt, excitement). Further, in previous work on personal narratives, McAdams and colleagues [46] assessed six of these specific emotions in evaluating the affective tone of personal narratives, and Coifman and colleagues [16] assessed three of these emotions in evaluating bereavement responses.

In the present study, McAdams and colleagues' [46] presence/absence coding system and method for calculating inter-rater reliability were employed. Raters were trained to rigorous standards of inter-rater reliability on a subset of the narratives (roughly 10% of the entire sample was coded by both raters), after which the remaining narratives were assessed independently by one rater. Periodic checks to prevent drift in the coding process were conducted. Inter-rater reliabilities were as follows: happiness: 0.86, surprise: 0.82, sadness: 0.89, fear/anxiety: 0.89, anger: 0.80, shame: 0.80, guilt: 0.82. Following standard narrative coding procedures, every narrative was coded for each theme sequentially, meaning that each of the 518 narratives was read eight separate times, resulting in 4,144 individual narrative codes.

### Analytical approach

The primary analytical strategy applied fixed-effects hierarchical linear modeling (HLM) to assess the growth curves of variables over time [47]–[49]. An advantage of HLM is its ability to account for missing data, participants with differing numbers of assessment points, and uneven spacing in the data collection schedule, collectively referred to as “unbalanced data” [47] (p. 146). Because the present study assessed change in a naturalistic treatment setting, the dataset is characterized by such irregularities, and it was therefore vital to have an analytic strategy that could accommodate such “messy” data. In each model, both the final estimation of fixed effects of the individual parameters will be presented (with robust standard errors), as well as the random effects for the overall model. Also, in every model, the passage of time is counted in number of days from the first assessment point, thus tapping the actual amount of time elapsed between assessments.

## Results

### Preliminary analyses

Before testing the primary hypothesis of the present study – that mixed emotional experience would be associated with improvement in psychological well-being over time – it was first necessary to assess the trajectories of participants' psychological well-being itself, as well as the association between each of the eight specific emotions coded in participants' narratives and psychological well-being over time. Indeed, if participants' overall psychological well-being did not reliably change over time, or if no significant change in a given emotion was associated with clinical improvement, then the data would be unable to speak to the primary hypothesis.

Results of this first set of models are displayed in Table 3. The first model (Model 1) was designed to assess whether participants' psychological well-being improved over time. In this model, the passage of time is entered at the first level as a predictor and psychological well-being is entered as the outcome. The results of this model indicate that, indeed, participants' psychological well-being did improve over the passage of time (aligning with the findings from decades of research indicating that psychotherapy is effective).

**Table 3:** Models of change in psychological well-being and specific emotions over time (final estimation of fixed effects, with robust standard errors).

Parameter	Coefficient	*t*	SE	Variance Component	SD	χ^2^	Deviance^a^
**Model 1: Change in Psychological Well-being over Time**							
**Intercept**	44.29	47.99**	0.93	17.44	4.18	859.14**	2753.04
**IPS Slope**	0.89	4.15**	0.21				
**Model 2: Happiness and Psychological Well-being**							
**Intercept**	44.28	31.82**	1.42	18.14	4.25	336.31**	1450.97
**# Days Slope**	0.05	5.21**	0.01				
**Happiness Slope**	1.91	1.79*	0.67				
**Model 3: Excitement and Psychological Well-being**							
**Intercept**	46.01	43.83**	1.05	18.56	4.31	546.24**	2237.03
**# Days Slope**	0.48	5.50**	0.09				
**Excitement Slope**	−0.82	−1.80	0.45				
**Model 4: Surprise and Psychological Well-being**							
**Intercept**	45.34	44.90**	1.01	18.59	4.31	546.30**	2240.09
**# Days Slope**	0.48	5.54**	0.09				
**Surprise Slope**	−1.78	−1.42	0.83				
**Model 5: Sadness and Psychological Well-being**							
**Intercept**	45.59	38.86**	1.17	18.17	4.26	339.22**	1451.21
**# Days Slope**	0.05	7.35**	0.01				
**Sadness Slope**	−1.28	−1.90*	0.68				
**Model 6: Fear/Anxiety and Psychological Well-being**							
**Intercept**	45.22	43.81**	1.03	18.59	4.31	543.40**	2242.04
**# Days Slope**	0.47	5.41**	0.09				
**Fear/Anxiety Slope**	−0.45	−0.88	0.52				
**Model 7: Anger and Psychological Well-being**							
**Intercept**	45.36	44.98**	1.01	18.62	4.31	541.81**	2242.14
**# Days Slope**	0.47	5.45**	0.09				
**Anger Slope**	−0.42	−1.19	0.35				
**Model 8: Shame and Psychological Well-being**							
**Intercept**	45.45	44.61**	1.02	18.74	4.44	540.21**	2231.89
**# Days Slope**	0.47	5.24**	0.09				
**Shame Slope**	−0.11	−0.15	0.77				
**Model 9: Guilt and Psychological Well-being**							
**Intercept**	45.45	44.89**	1.01	18.73	4.33	540.07**	2230.65
**# Days Slope**	0.47	5.45**	0.08				
**Guilt Slope**	−0.50	−0.57	0.88				
**Model 10: Concurrent Happiness and Sadness and Psychological Well-being**							
**Intercept**	45.26	31.75**	1.42	17.65	4.20	347.38**	1516.02
**# Days Slope**	0.05	4.66**	0.01				
**Mixed Emotions Slope**	1.29	2.89**	0.42				
**Model 11: Concurrent Happiness and Sadness and Psychological Well-being Controlling for Neuroticism**							
**Intercept**	44.77	28.10**	1.15	17.82	4.22	335.15**	1446.04
**# Days Slope**	0.05	5.27**	0.01				
**Neuroticism Slope**	−0.20	−0.22	0.92				
**Mixed Emotions Slope**	1.14	2.94**	0.45				

Note:

** p<.01,

* p<.05;

a all df = 46.

In the next set of models (Models 2–9), the relationship between each specific emotion and psychological well-being was assessed, accounting for the passage of time. In these models, specific emotion was entered at the first level, along with the passage of time as a simultaneous predictor, and psychological well-being was entered as the outcome variable. There was no second level to these models, as we were investigating trends across all participants, not looking for differences between sub-sets of participants. Models 2–9 therefore describe the relationships between each specific emotion and overall psychological well-being, accounting for the passage of time. These models suggest that increases in happiness and decreases in sadness were significantly associated with improvement in psychological well-being over time. None of the other specific emotions were significantly associated with improvement in psychological well-being.

#### Operationalizing mixed emotions

Given that happiness and sadness were the only specific emotions to show a significant relationship with psychological well-being over time, the six other specific emotions were dropped from subsequent analysis and a composite variable, representing instances when happiness and sadness co-occurred, was created. The remaining analyses investigated the unfolding relationship between the co-occurrence of happiness and sadness and psychological well-being. Indeed, recent empirical work has demonstrated that these positive and negative emotions are not necessarily opposite ends of a single continuum [50] and that both happiness and sadness can be experienced at the same time [19], [51]. In line with previous research that has focused on happiness and sadness specifically [18], [19], including Lazarus' proposal that these are emblematic positive and negative emotions [20], the present study adopted an empirical approach to the operationalization of mixed emotional experience. The two examples of narratives drawn from the present sample included in the Method section above both illustrate narratives that featured concurrent happiness and sadness.

### Primary analyses

#### Association between mixed emotions and psychological well-being

As described above, a composite variable capturing only those instances wherein participants' narratives were characterized by concurrent happiness and sadness was created. This allowed for the construction of a model testing the primary hypothesis of the present study: that concurrent happiness and sadness would be positively associated with psychological well-being over time. Model 10, as displayed in Table 3, was constructed with the composite mixed emotions variable and the passage of time as concurrent predictors and psychological well-being as the outcome. This model indicates that the concurrent experience of happiness and sadness was significantly positively associated with improvements in psychological well-being, controlling for the impact of the passage of time.

#### Controlling for relevant personality traits

Having provided support for the primary hypothesis of this study, it was also possible to assess the relationship between concurrent happiness and sadness and psychological well-being over time, controlling for the impact of dispositional personality traits that might explain this relationship. Specifically, the traits of neuroticism and extroversion have shown strong associations with affective experience and well-being [37], [38]. Before doing so, the overall change in each personality trait was assessed. Neuroticism was the only personality trait to show significant changes over the twelve assessment points (*t*(46) = −3.00, *p*<.01, Cohen's *d* = 0.88). While personality traits would not typically be expected to change significantly over the course of a roughly fourteen-week period (the mean period of participation in the present study) previous research has consistently found that certain trait scores, especially neuroticism, might be elevated during episodes of psychopathology [52] and some studies have observed shifts in traits over the course of psychotherapy independent of changes in psychopathology [53], [54].

Thus, the relationship between concurrent experience of happiness and sadness and psychological well-being was assessed controlling for the passage of time and for changes in trait neuroticism. This model, Model 11 in Table 3, replicated Model 10, but added a second level to the model, allowing it to examine whether there were significant differences in the relationship between mixed emotional experience and psychological well-being over time across participants with different levels of change in their trait neuroticism. Model 11 indicates that the association between mixed emotional experience and psychological well-being remained significant when additionally controlling for the passage of time as well as changes in trait neuroticism (indeed, the significant change in neuroticism itself was no longer significantly associated with changes in psychological well-being when the passage of time was included in the equation). In addition, a model testing the relationship between mixed emotional experience and psychological well-being also remained significant when Time 1 neuroticism was entered at a second level. In other words, there were no significant differences in the association between the concurrent experience of happiness and sadness and psychological well-being across participants with different levels of neuroticism before treatment. This pair of findings indicates that the association between experiencing happiness and sadness concurrently and improvements in psychological well-being is quite robust and cannot simply be reduced to the impact of long-standing dispositional personality traits associated with emotion and well-being.

#### The prospective link between mixed emotions and psychological well-being

Having established the association between one type of mixed emotional experience and improvements in psychological well-being it was also possible to investigate whether changes in mixed emotional experiences precede improvements in psychological well-being. Given the naturalistic setting of the present study it was not possible to determine causality; additional experimental manipulations would be required for that. However, association and temporal precedence are preconditions for causality. Having established a significant association between mixed emotional experience and psychological well-being we sought to examine these variables' temporal relationship. If the temporal precedence of changes in mixed emotional experience were observed, it would underscore the power of mixed emotional experience in influencing psychological well-being. Following recommendations by Singer and Willett [47], a model (displayed in Table 4) was constructed in which improvements in psychological well-being were lagged one assessment point and included in the model at level 1, such that the temporal precedence of changes in mixed emotional experience could be investigated. It has been suggested that this analytic strategy of using time-lagged HLM may offer insight into the causal relationship between variables [55]. This model (Model 12) indicated that there was a significant relationship between the concurrent experience of happiness and sadness and subsequent improvements in psychological well-being one assessment point later. A test of this model, controlling for changes in trait neuroticism remained significant (Model 13). Likewise, a multi-level test of this model, with neuroticism entered as a level-2 variable produced analogous results. These findings indicate that changes in mixed emotional experience were significantly positively associated with subsequent improvements in psychological well-being, controlling for the impact of dispositional traits. It is worth noting that a converse model (Model 14), in which changes in psychological well-being were assessed relative to changes in mixed emotional experience at a lag of one assessment point, was not significant.

**Table 4:** Additional models (final estimation of fixed effects, with robust standard errors).

Parameter	Coefficient	*t*	SE	Variance Component	SD	χ^2^	Deviance^a^
**Model 12: Concurrent Happiness and Sadness and Psychological Well-being at a Lag of 1 Assessment point**							
**Mixed Emotions Intercept**	0.05	0.19	0.28	0.36	0.60	30.89*	479.27
**Mixed Emotions Slope**	0.02	2.65**	0.01				
**Model 13: Concurrent Happiness and Sadness and Psychological Well-being at a Lag of 1 Assessment point Controlling for Neuroticism**							
**Mixed Emotions Intercept**	0.04	0.17	0.27	0.36	0.60	29.13*	479.80
**Neuroticism Slope**	−0.07	−0.95	0.08				
**Mixed Emotions Slope**	0.02	0.01**	0.01				
**Model 14: Psychological Well-being and Concurrent Happiness and Sadness at a Lag of 1 Assessment point**							
**STIC IPS Intercept**	47.11	35.50**	1.33	17.26	4.15	342.65**	1392.55
**STIC IPS Slope**	0.23	0.50	0.48				
**Model 15: Mixed Emotions and Psychological Well-being at a Lag of 1 Assessment Point Controlling for Happiness and Sadness Independently**							
**Mixed Emotions Intercept**	46.28	45.67**	1.01	20.20	4.49	483.53**	2077.15
**Happiness Slope**	0.98	1.98*	0.49				
**Sadness Slope**	−1.00	−1.85*	0.59				
**Mixed Emotions Slope**	1.42	2.87*	0.41				
**Model 16: Mixed Emotions and Psychological Well-being Controlling for Happiness and Sadness Independently**							
**Mixed Emotions Intercept**	47.40	48.40**	0.98	19.57	4.42	448.93**	1962.82
**Happiness Slope**	1.01	1.57*	0.57				
**Sadness Slope**	−1.17	−1.17*	0.49				
**Mixed Emotions Slope**	0.52	1.05	0.34				

Note:

** p<.01,

* p<.05;

a all df = 46.

#### Controlling for the independent effects of happiness and sadness

This relationship between concurrent happiness and sadness and subsequent improvements in psychological well-being remained significant when controlling for changes in happiness and sadness independently by entering them as concurrent predictors at level 1 of the model (Table 4, Model 15). However, this was not the case when predicting current psychological well-being status from concurrent happiness and sadness while controlling for happiness and sadness independently (Table 4, Model 16). In other words, the concurrent experience of happiness and sadness prospectively preceded increases in psychological well-being above and beyond the influence of happiness and sadness independently, but this effect was non-significant when examined at the same moment in time. The pattern of findings indicates that increases in the concurrent experience of happiness and sadness temporally precede increases in psychological well-being (and not vice versa). In addition, it suggests that the true benefit from concurrent happiness and sadness may not be instantaneous, but prospective, in line with theoretical predictions from the co-activation model of health [4].

## Discussion

The present study provides empirical evidence that mixed emotional experience is associated with and has a prospective positive relationship with psychological well-being in the context of psychotherapy. The results indicate that participants who experienced a concurrent mixture of happiness and sadness during the course of treatment enjoyed subsequent improvements in their psychological well-being. This finding remained significant when controlling for the impact of the passage of time as well as that of dispositional personality traits associated with affect. In addition, the results suggest that the significant association between the experience of concurrent happiness and sadness is uniquely related to psychological well-being at the following assessment point, but not concurrently, when controlling for the independent impacts of happiness and sadness themselves. In other words, mixed emotional experience was seen to have a prospective influence on psychological well-being, but its concurrent association with psychological well-being was explained by the independent effects of happiness and sadness. This suggests that mixed emotional experience may have a distinct prospective potency; its association with psychological well-being unfolds over time. Thus, while the concurrent experience of happiness and sadness in the face of adversity might not provide immediate benefit, it may signal enhancements in psychological well-being in the near future.

In the present study we employed a bottom-up, empirical approach to operationalizing mixed emotions. As a result, the current research also demonstrates specificity with respect to the relationship between mixed emotional experience and improvements in psychological well-being. Only the blend of happiness and sadness preceded enhanced psychological well-being, indicating that any mix of positive and negative emotions may not have the potency of concurrent happiness and sadness. Further, it is also worth noting that despite the voluminous literature on psychotherapeutic change [29], [31], [56] and the role of affect in psychotherapy [6], [56], a novel aspect of the present study is that it specifically examines the concurrent experience of positive and negative emotions as it relates to improvements in psychological well-being over the course of psychotherapy.

Importantly, the research paradigm that we employed never directly asked participants to report their emotional experiences; rather we were able to unobtrusively assess the emotions that participants were feeling by using a narrative coding procedure. This approach was grounded in work on patients' perspectives of treatment [26], [27], [41], where it has been fruitfully used for accessing individuals' private understanding of their experiences. In the context of the present study, such an approach allowed us to assess emotional experience in an especially ecologically valid fashion; participants were not aware that we would specifically be assessing their emotional experience and thus their narratives were not written in a way that might have purposely highlighted emotional content.

### Limitations

Our research was conducted in a naturalistic setting (an outpatient clinic), which speaks to the ecological validity of the study. However, the extent to which our findings can be generalized to non-therapy patients is currently unknown. Presumably, because our participants were demographically similar to the general treatment-seeking population [32] we can only offer speculation that the link between concurrent happiness and sadness and improvements in psychological well-being may be a generalizable phenomenon, at least within clinical populations, but future research will need to be conducted to fully determine this.

Along similar lines, it is possible that psychotherapy is a context in which negative emotions are particularly important. Certainly several approaches to psychotherapy, such as exposure-based treatments [28], specifically embrace negative emotional experience as vital to treatment outcome. In addition, newer positive psychotherapy approaches have explicitly focused on the different roles of negative and positive affect in psychotherapeutic outcome [22], but whether the present findings might apply in that domain is unknown. Thus, whether we would observe the effects that we found in specific treatment approaches or in non-therapeutic settings is still unknown. The inclusion of a non-clinical control group in our study would have facilitated such an extrapolation of the results. Future research should test whether mixed emotional experience benefits psychological well-being in non-clinical settings.

Using a similar approach to the one taken in several previous studies [16], [46], we examined the presence of several specific emotions in the course of participants' therapeutic process. Having found that happiness and sadness were the sole emotions that were linked to psychological well-being in our sample, we explored the relationship between the concurrent experience of these two emotions and improvement in psychological well-being. It is possible, of course, that other measures that tap into different aspects of emotional experience, such as arousal (e.g., the PANAS [57]) could yield different results, or at the very least, a more nuanced portrait of the relationship between mixed emotions and psychological well-being.

Further, the timeline of our study does not allow us to assess the long-term effects of mixed emotions on psychological well-being, or their relationship with treatment outcome. It is possible that the mixed emotions that our research participants experienced marked the beginning of a path of improvement, a marker of impending clinical outcomes as opposed to a mechanism, or it could be the case that mixed emotions are indicative of a more sustained high level of psychological well-being. Our investigation focused on identifying whether there was a prospective relationship between concurrent happiness and sadness and psychological well-being and used psychotherapy as the context for doing so. However, since the study only covered the initial phase of treatment it is possible that the longer-term relationship between mixed emotional experience and psychological well-being might look different.

It is also possible that the study design itself, with such frequent assessments, may have influenced the outcomes of the study. Although it is a problem common to longitudinal studies that include fine-grained assessment of variables over time, practice effects of repeated completion of the STIC may have biased subsequent responding. In addition, it is possible that the writing of the narratives may itself have acted as an intervention, enhancing psychological well-being. The ameliorative effects of written disclosure of difficult experiences are well-documented [8], [9]. The addition of a control group completing a writing exercise on non-emotional content would have enabled the present study to assess the impact of the narrative task itself.

The exact mechanism through which the concurrent experience of happiness and sadness is associated with psychological well-being is still unknown. Based on Larsen and colleagues' [4] co-activation model, we speculate that participants who experience concurrent happiness and sadness through the course of therapy may be, in essence, making meaning of negative events in their lives. That is, by experiencing happiness and sadness concurrently, individuals may theoretically be able to confront and process the events that ultimately led to sadness or negativity [10]. Although narrative construction has been previously been used as a vehicle to tap meaning-making [39]–[41], [58], future work should more directly measure whether meaning-making is a mediating link between mixed emotions and well-being.

Finally, the results of the current study essentially provide evidence for two of the three criteria necessary for causality [59]: co-variation and temporal precedence. Further research, however, should attempt to tackle the third criterion – the clean, confound-free manipulation of the independent variable – to rule out alternative explanations for cause-effect linkages. For example, a group of psychotherapy patients could be experimentally encouraged to approach stressors with a blend of happiness and sadness while others do not engage in this manipulation. Future research could also seek to address this limitation by extending our approach beyond the psychotherapeutic context to experimentally manipulate the concurrent experience of happiness and sadness and assess its impact on well-being in other settings.

### Implications for Future Research and Clinical Practice

This study has important implications for research on mixed emotional experience. In specifying a particular blend of emotions – concurrent happiness and sadness – that are associated with improved psychological well-being, the results of this study indicate that future research ought to focus on these specific emotions, or adopt an equally empirical approach to defining mixed emotional experience. Furthermore, the present study also suggests that future research on mixed emotional experience and well-being would benefit from study designs that allow for the fine-grained assessment of the dynamic course of this relationship over time.

Given the context in which it was conducted, this study also has important implications for both the study and practice of psychotherapy. Our findings indicate that fostering mixed emotional experience in psychotherapy clients may prospectively enhance improvements in their psychological well-being. This finding aligns with theory and results from the study of psychotherapy more generally [6], as well as some more recent promising work from the study of positive psychotherapy in particular [21]–[23]. Like these studies, the current research indicates that treatment should not focus strictly on the elimination of negative emotional experience, as negative emotions may be fundamental in the process of clinical improvement. Embracing the benefits of mixed emotional experience could be easily folded into most theoretical approaches to conducting psychotherapy.

### Conclusion

Taken together, the results of the present study suggest that experiencing happiness and sadness concurrently is associated with psychological well-being and that doing so temporally precedes improvements in psychological well-being among psychotherapy patients. Future work will hopefully clarify the generalizability of these findings, as well as the specific mechanisms by which mixed emotions can benefit psychological well-being. Nonetheless, the current work indicates that the co-activation of these specific positive and negative emotions – happiness and sadness – may provide the brightest path in the course of navigating life's challenges.
